# Above the dose threshold: a simple way to improve the delivery efficiency of nanomedicine

**DOI:** 10.1038/s41392-020-00400-7

**Published:** 2020-11-20

**Authors:** Lei Yang, Yanqi Ye, Lulu Cai

**Affiliations:** 1 Personalized Drug Therapy Key Laboratory of Sichuan Province, Department of Pharmacy, Sichuan Provincial People’s Hospital, University of Electronic Science and Technology of China, Chengdu, 610072 China; 2 Zenomics Inc., California NanoSystems Institute, University of California, Los Angeles, California, 90095 USA

**Keywords:** Drug development, Drug development

Recently, a paper by Warren Chan et al. published in Nature Materials, discovered a simple way to improve the tumor delivery efficiency by reaching a nanomedicine dose above a threshold.^1^ As the largest organ of the reticuloendothelial system, the liver takes up majority of nanomedicine after ingestion.^2^ This dose threshold is based on the kinetic balance between the uptake of nanomedicine in the liver and the absorption in the tumor, which is related to the number of receptors and binding sites available on Kupffer cells residing in the liver (Fig. 1). A dose exceeding the effective binding site threshold overwhelmed the Kupffer cells, therefore it can reduce the liver clearance of nanomedicine and prolong the circulation.

**Fig. 1:**
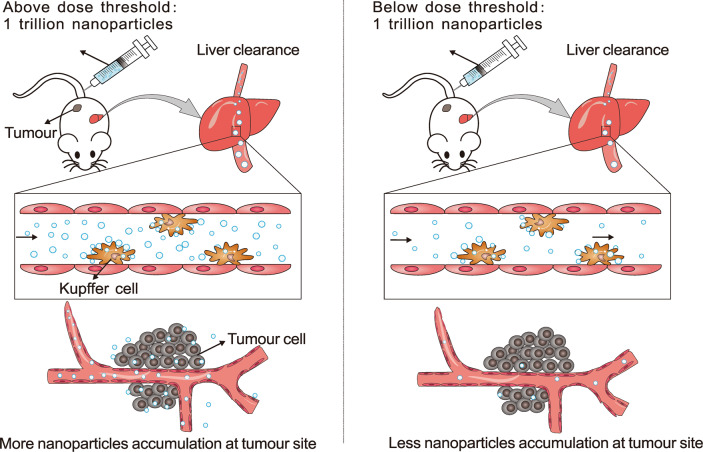
Above the dose threshold: Large numbers of nanoparticles above the 1 trillion threshold overwhelmed Kupffer cells, reduced liver clearance, prolonged circulation and enhanced accumulation in the tumor

To investigate the mechanisms, three magnitude orders (50 billion to 50 trillion) of nanoparticles were injected into 4T1 tumor-bearing BALB/c mice. Chan et al. found that the plasma half-life of nanoparticles extended as the dose increased, demonstrating that high-dose could significantly reduce the hepatic clearance. In addition, after a single dose of more than 1 trillion, the uptake of nanoparticles by Kupffer cells was restricted within 24 h, which was unrelated to Kupffer cell death nor accelerated blood clearance (ABC) phenomenon.

Next, the authors observed that the uptake efficiency of nanoparticles by Kupffer cells did not reach saturation through in vivo imaging, demonstrating the major factor limiting the liver clearance was uptake rate instead of uptake capacity. Subsequently, the author studied three uptake pathways of general Kupffer cells: clathrin- and caveolin-mediated endocytosis, macropinocytosis and receptor-mediated phagocytosis. They found the dose threshold of nanoparticles was correlated with the number and binding sites of receptors available on Kupffer cells, although the specific receptors remain to be determined.

Furthermore, the authors investigated the relationship between the dose threshold and tumor accumulation of nanoparticles. They have found that dose augment of nanoparticles can significantly increase the number of particles at the tumor site, resulting from the overwhelmed liver enrichment. The authors also compared the biodistribution of a single dose above the threshold to multiple doses below the threshold with the same total dose. The results showed that although the total numbers of nanoparticles in the two groups were the same, the drug accumulation in the liver was lower after the single high-dose administration, leading to longer circulating half-life and more efficient tumor delivery. In addition, the authors chose benign filler nanoparticles and increased the number of nanoparticles without increasing the dose of active drugs. The 4T1 tumor-bearing BALB/c mice with an additional dose of administration enhancer were compared to the control group that received the same dose of therapeutic nanoparticles of more than 1 trillion. They found that in the experimental group, the doxorubicin levels in serum and tumor were higher evidenced by doxorubicin-positive nuclei. The tumor volume was reduced by 57%, and the survival time was extended by 29%. Overall, the treatment effect was significantly better than the control group.

Finally, the authors conducted literature research on the clinical use of nanomedicine. The author recalculated the dose of nanoparticles in 40 publications and obtained 67 total doses and dosing data points. They found that the dose calculated by the number of nanoparticles was related to the tumor delivery. The median dose used in these papers was 1.2 trillion nanoparticles, which was close to the 1 trillion thresholds observed in the experiments. There are significantly more documents with doses above this threshold than those with doses below this threshold. These results emphasize the important relationship between quantitative nanoparticle doses and tumor treatment.

In summary, the study by Chan and colleagues discovered that the 1 trillion dose threshold can enhance the delivery of nanoparticles to improve the therapeutic effeciency. This specific value may vary depending on the design of the nanoparticle and the delivery target, but it forms the foundational dose threshold concept of more than 1 trillion nanoparticles. Extensive and careful assessment of nanoparticle dose threshold will provide a powerful, versatile, yet simple direction to translational drug delivery strategies.

## References

[1] Ouyang, B. et al. The dose threshold for nanoparticle tumour delivery. *Nat. Mater.*10.1038/s41563-020-0755-z(2020). 10.1038/s41563-020-0755-z 32778816

[2] Zhang YN (2016). Nanoparticle-liver interactions: Cellular uptake and hepatobiliary elimination. J. Controlled Release..

